# Role of Polyoxometalate Contents in Polypyrrole: Linear Actuation and Energy Storage

**DOI:** 10.3390/ma15103619

**Published:** 2022-05-18

**Authors:** Quoc Bao Le, Zane Zondaka, Madis Harjo, Ngoc Tuan Nguyen, Rudolf Kiefer

**Affiliations:** 1 Conducting Polymers in Composites and Applications Research Group, Faculty of Applied Sciences, Ton Duc Thang University, Ho Chi Minh City 700000, Vietnam; lequocbao@tdtu.edu.vn; 2 Intelligent Materials and Systems Lab, Faculty of Science and Technology, University of Tartu, Nooruse 1, 50411 Tartu, Estonia; zane.zondaka@ut.ee (Z.Z.); madis.harjo@gmail.com (M.H.); 3 Faculty of Applied Sciences, Ton Duc Thang University, Ho Chi Minh City 700000, Vietnam; nguyenngoctuan@tdtu.edu.vn

**Keywords:** PTA concentration, PPy/DBS-PT films, linear actuation, organic electrolyte, specific capacitance

## Abstract

A combination of polyoxometalates with polypyrrole is introduced in this work. Our goal was to include phosphotungstic acid (PTA) in different molar concentrations (0.005, 0.01, and 0.05 M) in the electropolymerization of pyrrole doped with dodecylbenzene sulfonate (DBS) and phosphotungstinates (PT), forming PPy/DBS-PT films. Scanning electron microscopy (SEM) revealed that the PPy/DBS-PT films became denser and more compact with increasing PTA concentrations. The incorporation of PT in PPy/DBS was analyzed using Fourier-transform infrared (FTIR) and energy dispersive X-ray (EDX) spectroscopy. The linear actuation in cyclic voltammetry and potential square wave steps in an organic electrolyte revealed increasing mixed actuation, with major expansion upon oxidation found for PPy/DBS-PT films with a PTA concentration of 0.005 M. Best results of a strain of 12.8% and stress at 0.68 MPa were obtained for PPy/DBS-PT (0.01 M). The PPy/DBS-PT films polymerized in the presence of 0.05 M of PTA and showed main expansion upon reduction, changing the actuation direction. Chronopotentiometric measurements of PPy/DBS-PT samples were conducted to determine the specific capacitance optimal for a 0.01 M PTA concentration in the range of 80 F g^−1^ (±0.22 A g^−1^).

## 1. Introduction

Polyoxometalates (POMs), known as metal oxide cluster anions [1], have been found beneficial due to their catalytic and redox reactive functionality. There are different types of POMs that are different in their structure, with most applying the Kegging-type structure [2] in the general formula of PM_12_O_40_^3−^, where the transition metal, M, can be tungsten (W) or molybdenum (Mo). The central group is tetrahedral PO_4_ units surrounded by twelve MO_6_ octahedral clusters [1], leading to a high surface area. POMs have high oxidation states, fast multiple reversible electron transfer reactions, and consistency in their structure. These nanostructured clusters are excellent candidates for electrochemical applications, such as sensors [3], solar cells [4], catalysts [5], and energy storage devices [6,7,8]. Our focus is on the combination of conducting polymers with Kegging-type POMs (phosphotungstic acid (PTA), PW_12_O_40_^3−^), which can either be the dopant in electropolymerization [9,10] or be covalently attached to conducting polymers [11,12]. POMs are soluble in aqueous solution; therefore, electropolymerization is performed by adding PTA as an extra dopant, forming PPy/DBS-PT [13] composites (PT^4−^ phosphotungstinate anions). Only a few studies have investigated the addition of POM in PPy in view of linear actuation, with most cases conducted on aqueous electrolytes, and recent research [14] shows that one of the best linear strains was 20% for PPy/DBS-PT in a PTA concentration of 0.01 M. The best linear strain for PPy/DBS in the aqueous electrolytes was found in the range of 12% [15]. PPy/DBS is a specific type of cation-driven actuator, where the large immobile DBS^−^ anions balance the positive charge upon oxidation, and upon reduction the left negative charge is compensated with cations and solvents (including osmotic pressure [16]) of the surrounding electrolytes [17]. If those PPy/DBS are applied in the organic electrolyte, the immobile DBS^−^–cation^+^ pairs cannot dissociate, and the formerly cation-driven PPy/DBS becomes anion-driven [18,19], with new places of PPy charged upon oxidation, followed by anion ingress. It was found from recent research [20] that PPy/DBS-PT films have also shown expansion upon oxidation with a similar solvent effect, whereby PT^4−^ cation complexes cannot dissociate in organic electrolytes. The mixed actuation revealed in former research was the reason that some anions that were incorporated at charging of PPy/DBS-PT films became immobile, and upon reduction, they were compensated with cations of the surrounding electrolytes.

Our goal in this work was to determine how PPy/DBS-PT with different PTA concentrations ranging from 0.005 to 0.05 M affects the actuation properties in an organic electrolyte (TBAPF_6_-PC). This novel approach focuses on how different PTA concentrations in electropolymerization influence the PPy/DBS-PT films’ formation, the actuation response in view of strain and stress, and which actuation direction occurs.

Cyclic voltammetry and potential square wave steps at different frequencies (0.0025 to 0.1 Hz) of PPy-DBS-PT films were performed in combination with linear actuation of stress and strain. In most combinations of POMs with polypyrrole, the energy storage capability is investigated [7,21], which we also addressed in this work on PPy/DBS-PT films (with different PTA concentrations) by applying chronopotentiometry. The PPy/DBS-PT films were characterized by scanning electron microscopy (SEM), energy dispersive X-ray spectroscopy (EDX), and Fourier-transform infrared (FTIR) spectroscopy.

## 2. Materials and Methods

### 2.1. Chemicals

Sodium dodecylbenzenesulfonate (NaDBS, 99%), ethylene glycol (EG, 99.8%), tetrabutylammonium hexafluorophosphate (TBAPF_6_, >99%), phosphotungstic acid hydrate (PTA, PW_12_O_40_^3−^, reagent grade), and propylene carbonate (PC, 99%) were supplied from Sigma-Aldrich (Taufkirchen, Germany). Commercial Durapore (Millipore, 0.22 μm, Sigma-Aldrich) was used as supplied. Pyrrole (Py, 98%) from Sigma-Aldrich was distilled prior to use and stored at a low temperature (−20 °C) in the dark under a nitrogen atmosphere. Milli-Q+ (Tallinn, Estonia) deionized water was used as supplied.

### 2.2. Electropolymerization

The galvanostatic electropolymerization (0.1 mA cm^−2^, −20 °C, 11.1 h) occurred in a two-electrode cell with a stainless-steel working electrode (18 cm^2^) and a stainless-steel mesh counter electrode opposite it. The monomer solution consists of 0.1 M pyrrole, 0.1 M NaDBS, and different molar concentrations of PTA (0.005, 0.01, and 0.05 M). To obtain polymerization at a low temperature below the freezing point, the anti-freezing agent ethylene glycol (EG) was included with a mixture of EG:Milli-Q+ of 50:50 wt.%. Films of PPy/DBS and PPy/DBS-PT were peeled off the stainless-steel electrode, washed several times with ethanol to remove pyrrole residue, and washed with Milli-Q+ to remove excess NaDBS and unbound PTA. The films were dried in an oven at 60 °C (2 mbar) for 12 h. The PPy/DBS-PT films were found with a thickness (weight) of 22.1 ± 1.1 μm (240.0 ± 13.0 μg) for 0.005 M PTA, 20.4 ± 1.2 μm (226.0 ± 10.0 μg) for 0.01 M PTA, and 18.6 ± 0.9 μm (215.0 ± 9.8 μg) for 0.05 M PTA. The average weight of all film samples was calculated as 227.0 ± 12.2 μg. The PPy/DBS films were found with a thickness of 23.5 ± 1.2 μm, with a weight of 236.0 ± 12.6 μg.

### 2.3. Linear Actuation of PPy/DBS-PT Samples

The PPy/DBS-PT and PPy/DBS films were cut to a 1.2 cm length with a 0.2 cm width and stored for 24 h in TBAPF_6_-PC electrolyte. For linear measurements, a self-made linear muscle analyzer was applied [22], either addressing the change of mass (calculated to stress, a constant length between both clamps in the range of 3 mm) or the change of length (strain, constant force, 9.8 mN~0.5 MPa). The PPy/DBS-PT films were fixed, with one side on the force sensor (TRI202PAD, Panlab, Barcelona, Spain) and the other side clamped on a steady arm with gold contacts embedded in the 0.1 M TBAPF_6_-PC electrolyte in the three-electrode cell. The clamped PPy/DBS-PT films were connected as working electrodes, with a platinum sheet as a counter-electrode and an Ag/AgCl (3 M KCl) reference electrode. The films were stretched for 6 h at 1% in the linear muscle analyzer before measurements. The linear muscle analyzer consists of a movable stage where the films can be stretched to a certain length, and the stiffness factor, k (mg.μm^−1^, calibration for length change measurements), can be determined before and after actuation measurements. The linear muscle analyzer change of length or mass is directly connected to the potentiostat (Biologic PG581, Seyssinet-Pariset, France), with self-written software responding in real-time output at applied electrochemical techniques. Cyclic voltammetry (including the pristine PPy/DBS films) and chronoamperometric measurements (frequencies of 0.0025–0.1 Hz) were performed with PPy/DBS-PT films to evaluate the strain and stress responses in the applied potential range of 1.0 to −0.55 V. At each applied frequency, the diffusion coefficients of the PPy samples were determined following Equations (1) and (2) [23]:

(1) $$\ln\left(1-\frac{Q}{Q_{t}}\right)=-b\cdot t$$

(2) $$D=\frac{b\cdot h^{2}}{2}$$

The left terms of Equation (1) were obtained by integration of square potential time curves, obtaining the charge densities “*Q*” at each time, t, divided by the total charge density “*Q_t_*”, yielding a curve where from the initial time, the slope “*b*” can be determined. With the thickness, *h* (average of all applied PPy/DBS-PT films of 20 μm), the diffusion coefficients upon oxidation (D_ox_) and reduction (D_red_) were calculated.

To ascertain the specific capacitance (pseudo-capacitance) of PPy/DBS-PT films, the current densities, j (current, i/mass), were obtained in the following order: ±0.22 A g^−1^ (0.0025 Hz), ±0.44 A g^−1^ (0.005 Hz), ±0.88 A g^−1^ (0.01 Hz), ±2.2 A g^−1^ (0.025 Hz), ±4.4 A g^−1^ (0.05 Hz), and ±8.8 A g^−1^ (0.1 Hz). At each current density, the same charge density of ±44 C g^−1^ was obtained. From the potential time curves at discharging (after IR drop), the “*slope*” was obtained at each applied current density (i/m) of the PPy/DBS-PT films to calculate the specific capacitance (*C_s_*) (Equation (3)):

(3) $$C_{s}=\frac{i}{\left(-slope\cdot m\right)}$$

### 2.4. Characterizations

SEM images (VEGA TESCAN, Tescan Orsay Holdings, Czech Republic) of the surface and cross-section (broken in liquid nitrogen) of PPy/DBS-PT films (dried state) were taken directly after polymerization. FTIR spectroscopy (1800–800 cm^−1^, Bruker Alpha, with Platinum ATR, Billerica, MA, USA) of PPy/DBS-PT samples with pristine PPy/DBS films was performed. The PTA powder was pressed into KBr pellets. The ion content from the cross-section image of PPy/DBS-PT films after actuation cycles was investigated with EDX spectroscopy (Oxford Instruments with X-Max 50 mm^2^ detector, High Wycombe, UK) of films in the oxidized state (1.0 V) and the reduced state (−0.55 V). The electronic conductivity of PPy/DBS-PT films was measured using a four-point probe conductivity meter (RM2, Jandel 4-Point Probe Head, Leighton Buzzard, UK).

## 3. Results and Discussions

In general, PTA inclusion in PPy samples leads to enhanced capacitance [7] due to the higher amount of negative charges of PT^4−^ macro-anions provided that led, especially in the aqueous electrolyte, to an increase of linear actuation [13]. Here, we show the effect of PPy/DBS-PT films in an organic electrolyte while keeping in mind that pristine PPy/DBS changed the actuation direction from cation-driven in the aqueous electrolyte to anion-driven in an organic electrolyte [18,24]. Besides the applied solvent in electropolymerization [20], the potential window also plays a role in the actuation direction [25]. Here, we have chosen the potential window of 1.0 to −0.55 V, which shows, in most cases of pristine PPy/DBS films, the main expansion upon oxidation, while small expansion upon reduction was also observed depending on the applied electrolytes [26]. Each PPy/DBS-PT film at PTA concentrations of 0.005, 0.01, and 0.05 M formed at least three samples, independent from each film polymerized with linear actuation, shown as mean values with standard deviations. First, we present the characterization of the PPy/DBS-PT films, including the electropolymerization of the films as well as the material characterization, such as SEM images and FTIR spectroscopy. EDX spectroscopy of PPy/DBS-PT films was performed after actuation cycles (~1000 cycles per sample) to determine the ion content at the oxidized/reduced state.

### 3.1. Characterization of PPy/DBS-PT Films

**Figure 1:**
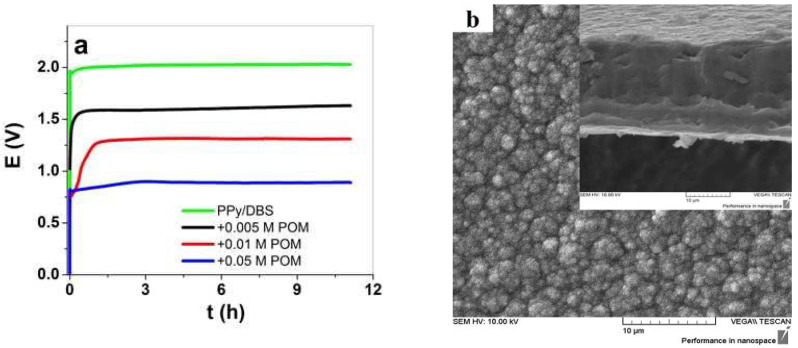

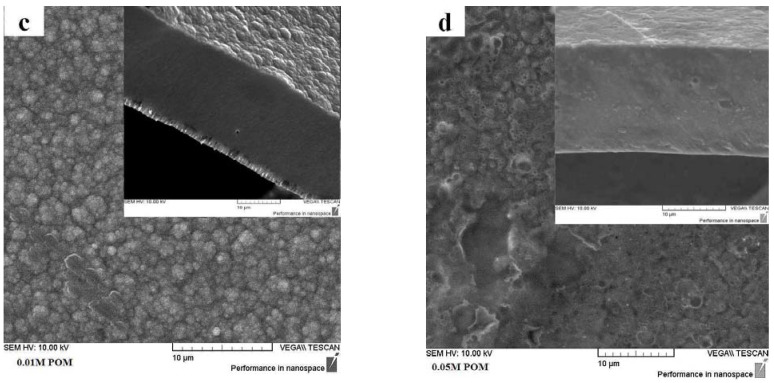
(**a**) Galvanostatic electropolymerization (0.1 mA cm^−2^) showing potential against time of PPy/DBS (green curve), with additions of 0.005 M PTA (black curve), 0.01 M PTA (red curve), and 0.05 M PTA (blue curve). The SEM surface images, with insets showing cross-section images (scale bar 10 μm), of PPy/DBS-PT films with different concentrations of PTA are presented in (**b**) with 0.005 M, (**c**) with 0.01 M, and (**d**) with 0.05 M.

The galvanostatic electropolymerization of PPy/DBS-PT films is presented in Figure 1a, and the SEM images of the surface and cross-section images are shown in Figure 1b–d. The SEM surface and cross-section images of pristine PPy/DBS are shown in Figure S1.

The electropolymerization curves of PPy/DBS and PPy/DBS-PT films are shown in Figure 1a, and it was revealed that the potential decreased with the increasing PTA concentration. At the end of polymerization, PPy/DBS had a potential of 2.03 V, PPy/DBS-PT (0.005 M) was found in the range of 1.64 V, PPy/DBS-PT (0.01 M) had a potential of 1.32 V, and the lowest potential of 0.87 V was found for PPy/DBS-PT (0.05 M). In general, upon electropolymerization, the first initial step is the oxidation of pyrrole monomers forming radical cations that combine with dimers until they become oligomers, and at a certain chain length, they become insoluble in the monomer solution and deposit on the stainless-steel working electrode [27,28]. The speed at which this takes place is influenced by the temperature, the electrolyte, or the solvent, which are essential parameters for obtaining dense, compact, and ordered films. The same polymerization condition was used in the choice of temperature and time for all PPy samples. The PTA itself is well-known as a mediator and a catalyst [2,10] that reduces the potential in electropolymerization. The SEM images of PPy/DBS-PT films in surface morphology (Figure 1b,c) and PPy/DBS (Figure S1) revealed the typical cauliflower structure [29]. The PPy/DBS-PT (0.05 M) film in surface morphology looked different, with less of a cauliflower structure and a smoother appearance than the other samples. The cross-section images shown in Figure S1 for PPy/DBS and in Figure 1b–d for PPy/DBS-PT films revealed that the films became denser and more compact with the increasing PTA concentration in electropolymerization. Table 1 shows the electronic conductivity and elastic modulus of PPy/DBS-PT films determined from the k factor obtained from the linear muscle analyzer.

The electronic conductivity and elastic modulus of PPy/DBS-PT films before (BA) and after actuation (AA) cycles (~1000 cycles) are shown in Table 1.

The electronic conductivity of PPy/DBS-PT films revealed an increase for the PTA concentrations 0.005 and 0.01 M after actuation cycles. Pristine PPy/DBS before actuation was found in the range of 6.6 ± 3.5 S cm^−1^, and it increased 1.3 times after actuation cycles, to 8.8 ± 4.7 S cm^−1^. The more compact the PPy/DBS-PT films become, the better the conductivity due to less distance between the charged chains [30]. The densest PPy/DBS-PT (PTA 0.05 M) in the inset in Figure 1d revealed (Table 1) that the surface conductivity was found optimal before actuation. After actuation, the conductivity in the case of PPy/DBS-PT (0.05 M) decreased 1.1 times, while all other PPy/DBS-PT samples showed an increase in the range of 1.3–1.5 times. The elastic modulus was obtained from the k factor in the linear actuation stage before actuation and showed an elastic modulus of PPy/DBS-PT films (Table 1) in the range between 0.82 and 2.45 MPa. The elastic modulus of PPy/DBS films before actuation was found in the range of 0.47 ± 0.02 MPa and increased to 0.98 ± 0.04 MPa after actuation.

After actuation, the elastic modulus decreased for PPy/DBS-PT films, as shown in [31], where additional carbide-derived carbon was included. In the case of PPy/DBS-PT films, the highest decrease was found for the 0.01 M PTA concentration in the range of a 4.3 times lower elastic modulus, while 0.005 M PTA had a 1.5 times reduction. The PPy/DBS-PT (0.05 M) was a brittle film reflected in the lowest elastic modulus at 0.82 MPa, with a 1.2 times decrease after actuation.

**Figure 2:**
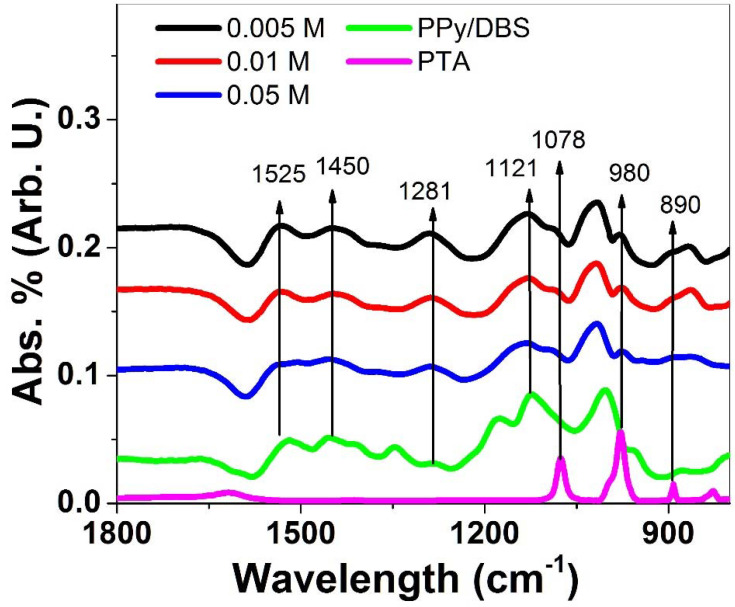
FTIR spectroscopy (1800–800 cm^−1^) of PPy/DBS-PT films with PTA concentrations of 0.005 M (black line), 0.01 M (red line), and 0.05 M (blue line) compared to pristine PPy/DBS (green line) and PTA (pink line).

Further characterization regarding FTIR measurements of PPy/DBS-PT films directly after polymerization in an oxidized state (~1.0 V), including pristine PPy/DBS films and PTA, were performed, and the results are presented in Figure 2.

**Figure 3:**
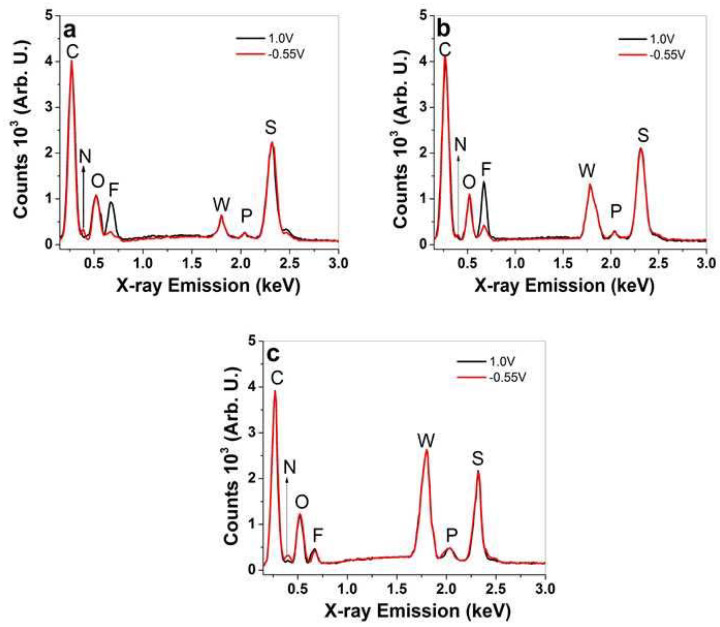
EDX spectroscopy of PPy/DBS-PT films (cross-section image) after actuation cycles upon oxidation (1.0 V, black line) and reduction (−0.55 V, red line), with different PTA concentrations of (**a**) 0.005 M, (**b**) 0.01 M, and (**c**) 0.05 M.

Typical PPy peaks [32,33,34] were found in all spectra (Figure 2), of 1525 cm^−1^ (C=C stretching vibration), 1450 cm^−1^ (C-N stretching vibration of PPy ring), 1281 cm^−1^ (C-N in-plane deformation shown in the literature at 1289 cm^−1^ [32]), and 1121 cm^−1^ (C-H bending modes). Additional peaks were not observed in PPy/DBS and were shown only in PPy/DBS-PT compounds reflected in the spectra of PTA, having bands at 1078 cm^−1^ that belong to P-O stretching vibrations, 980 cm^−1^ belonging to terminal stretching modes of W-O, and 890 cm^−1^, describing the vibration of W-O-W bonds [35]. The incorporation of PTA in PPy/DBS could be identified in FTIR analysis. Further investigations of the ion content of PPy/DBS-PT films after actuation in oxidized/reduced form are displayed in Figure 3a–c. The content of PPy/DBS-PT films directly after polymerization (oxidized state, ~1.0 V) is shown in Figure S2.

At all spectra of PPy/DBS-PT films (Figure 3a–c), certain elements were found that were not altered upon oxidation/reduction, such as carbon at 0.27 keV (C), oxygen at 0.52 keV (O), tungsten at 1.78 keV (W), phosphor at 2.04 keV (P), and sulfur at 2.32 keV (S). The differences in PTA (PW_12_O_40_^3−^) concentrations in PPy/DBS-PT films can be observed in Figure S2, revealing an increase of the tungsten peak at 1.78 keV with increasing PTA concentrations in electropolymerization. Comparing the intensities of this peak (Figure S2), it was found to be 1.68 times higher for 0.01 M PTA and 2.7 times higher in tungsten for 0.05 M of PPy/DBS-PT films, compared to 0.005 M PTA.

Upon oxidation/reduction, the PTA peaks revealed (Figure 3a–c) that there were no decreases observed, which was different compared to former research [9] in that the PT^4−^ anions were not removed in the redox reaction and maintained relatively constant amounts in the PPy network [36]. The changes of certain elements upon oxidation/reduction are shown in Figure 3a–c regarding the TBA^+^ cations reflected in the nitrogen peak at 0.4 keV (N) and the PF_6_^−^ anions shown in the fluoride peak at 0.67 keV (F), while the phosphor peak overlapped with the incorporated PT^4−^ anions. In the case of the nitrogen peak, a small increase was found with increasing PTA concentrations of PPy/DBS-PT samples upon reduction (−0.55 V). The change in fluoride signals that belongs to PF_6_^−^ anions for PPy/DBS-PT with PTA concentrations of 0.005 M (Figure 3a) and 0.01 M (Figure 3b) showed an increase in counts (anion ingress) upon oxidation, while a decrease was observed upon reduction (there was a minor fluoride signal still observed upon reduction). In case of PPy/DBS-PT with a 0.05 M PTA concentration, a fluoride signal upon oxidation is shown in Figure 3c which did not alter much upon reduction, leading to the assumption that a certain amount of PF_6_^−^ anions stayed inside the film. PTA itself cannot be dissolved in PC, and therefore we assume it is comparable to DBS^−^ as the solvent PC had a similar effect, and upon oxidation, new sites were formed that were compensated with PF_6_^−^ anions. A further investigation of how the different ion movements affect the performance of PPy/DBS-PT films was conducted using linear actuation studies, as shown in the next section.

### 3.2. Linear Actuation of PPy/DBS-PT Composites

Conducting polymers are faradaic actuators [37], where the charge determines the linear actuation response. To avoid an irreversible reaction, either over-oxidation or over-reduction, the potential range of 1.0 to −0.55 V was chosen, presenting a “steady-state” condition with charging/discharging in balance [38]. The actuation measurements included cyclic voltammetry and square wave potential steps to evaluate how different concentrations of PTA in electropolymerization influence the linear response of PPy/DBS-PT films under strain and stress. At least three different PPy/DBS-PT samples were taken from each polymerization measurement and presented as mean values and standard deviation.

#### 3.2.1. Cyclic Voltammetry

**Figure 4:**
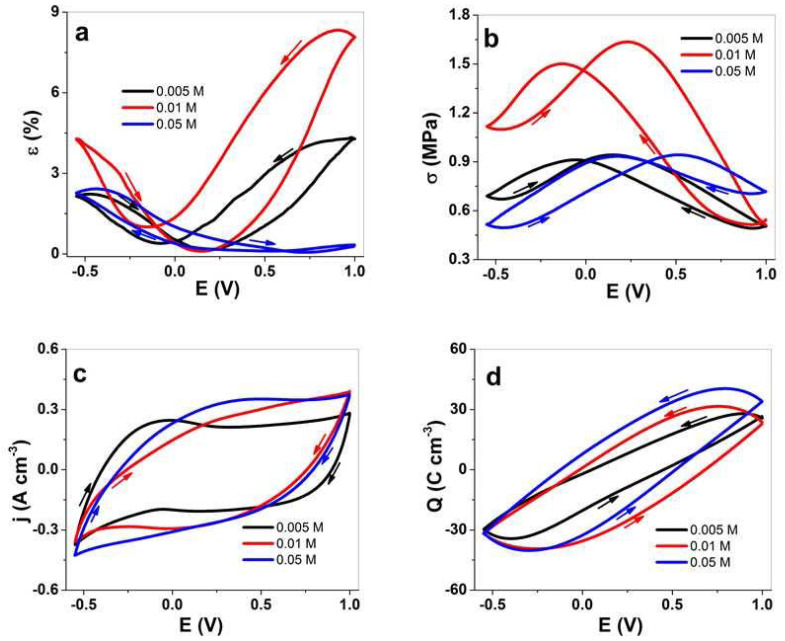
Cyclic voltammetry (scan rate 5 mV s^−1^, 3rd cycle) of PPy/DBS-PT films with PTA concentrations of 0.005 M (black line), 0.01 M (red line), and 0.05 M (blue line) in the TBAPF_6_-PC electrolyte. The strain, ε, is shown in (**a**), the stress, σ, in (**b**), the current density, j, in (**c**), and the charge density, Q, in (**d**) against the potential, E (1.0 to −0.55 V). The arrows show the direction of the scans.

PPy/DBS-PT with different PTA concentrations were investigated via cyclic voltammetry, with included measurements of linear actuation regarding strain and stress, as shown in Figure 4a,d, respectively. The current density potential curves are displayed in Figure 4c, and the cyclovoltammetry responses are shown in Figure 4d.

For PPy/DBS-PT films, the strain curves (Figure 4a) revealed mixed actuation with main expansion upon oxidation (minor expansion upon reduction) in the range of 4.2% (2.1% at −0.55 V) for 0.005 M PTA and 8.1% (4.2% at −0.55 V) for 0.01 M PTA. Surprisingly, the PPy/DBS-PT film with 0.05 M PTA showed main expansion upon reduction in the range of 2.3% (~0.3% expansion upon oxidation). The potential stress curves (stress is always opposite to strain, where contraction is reflected in expansion for strain) in Figure 4b showed a similar picture for all PTA concentrations of PPy/DBS-PT films having mixed-ion actuation. The PPy/DBS-PT (0.05 M) film’s main actuation direction was found to reduce stress in the range of 0.21 MPa upon reduction. The best stress difference (difference of stress upon oxidation and reduction) so far was found for PPy/DBS-PT with a PTA concentration of 0.01 M in the range of 0.6 MPa. Previous research [14] studying PTA concentrations in aqueous electrolytes revealed that PPy/DBS-PT with a PTA concentration of 0.01 M showed the best strain response, which agrees with the results of the current study.

The inclusion of PTA molecules in the PPy network, whereby the PT^4−^ anions are immobile, showed via EDX spectroscopy (Figure 3a–c) that no decrease of PT^4−^ anions took place during the reversible redox reaction. Additionally, with increasing PTA concentrations, the fluoride peak in EDX spectroscopy increased upon reduction, revealing that more PF_6_^−^ anions stayed in the PPy/DBS-PT films, which explains the increasing strain (Figure 4a) at discharge. One possible reason for such phenomena we assume relates to the more dense formation of PPy/DBS-PT films at higher PTA concentrations, which was also shown in previous research, where 0.1 M PTA was applied and mixed-ion actuation was observed [39]. The electrolyte TBAPF_6_ applied in the PC solvent has to be considered, with TBA^+^ cations with a van der Waals radius of 0.415 nm [40], not solvated [41], with ion conductivity in PC in the range of 9.1 S cm^2^ mol^−1^, and moving as a single entity in PPy/DBS-PT films. The PF_6_^−^ anions’ van der Waals radius was found in the range of 0.256 nm, with nearly double the ion conductivity at 17.9 S cm^2^ mol^−1^ [40], as well as being weakly solvated [42] and having a spherical form, and they moved faster in and out upon redox reactions than the larger TBA^+^ cations. It is well-known that regardless of the size, anions such as triflate (CF_3_SO_3_^−^) and PF_6_^−^ can become partly immobile in anion-driven PPy films with some extent of mixed actuation (expansion upon reduction), as shown from past research [43,44]. In the case of the TBAPF_6_-PC electrolyte in PPy/DBS films, the main actuation direction was upon oxidation with the strain of 3% (upon reduction 0.8%) and a stress difference of 0.83 MPa, as presented in Figure S3a,b, respectively.

The current density potential curves (CV curves) presented in Figure 4c revealed that the oxidation peaks of PPy/DBS-PT films were relatively weak, and shifted with higher concentrations of PTA during electropolymerization to a more positive potential. For PPy/DBS-PT films with a PTA concentration of 0.005 M, the oxidation peak was located at −0.05 V, which is comparable to pristine PPy/DBS films [26] (Figure S3c). The oxidation peak for PPy/DBS-PT films at 0.01 M PTA was found at 0.23 V, and 0.05 M PTA had an oxidation peak at 0.35 V. The positive shift of the oxidation peak shown from previous research regarding PTA inclusion in PPy was due to the catalytic activity of PTA [4]. The current density of PPy/DBS-PT films was larger with a higher amount of PTA, which is outlined in the charge density curves in Figure 4d. With increasing concentrations of PTA in PPy/DBS-PT films, the charge density increased and was found in the range of 56 C cm^−3^ for 0.005 M PTA, comparable to PPy/DBS (Figure S3d) with 56.5 C cm^−3^, 61 C cm^−3^ for 0.01 M PTA, and 65 C cm^−3^ for 0.05 M PTA.

#### 3.2.2. Square Wave Potential Steps of PPy/DBS-PT Films

**Figure 5:**
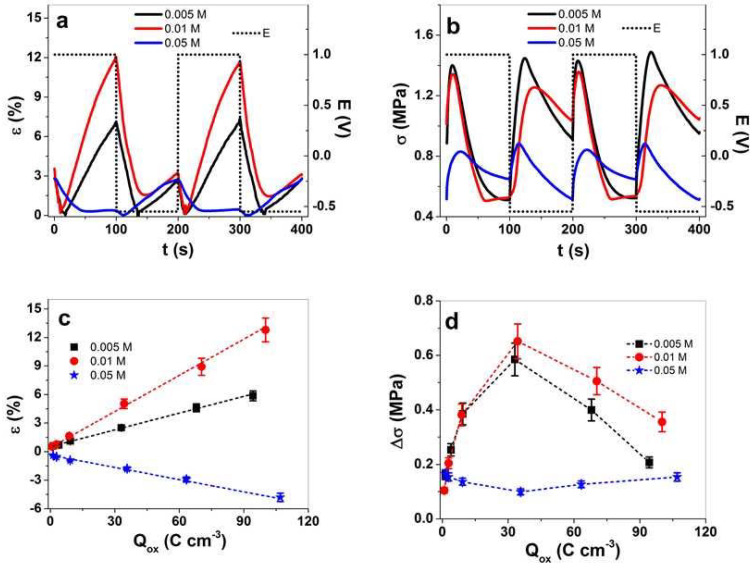
Square potential steps of PPy/DBS-PT films in the TBAPF_6_-PC electrolyte with different PTA concentrations during polymerization, such as 0.005 M (black line, ■), 0.01 M (red line, ●), and 0.05 M (blue line, 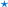), at a frequency of 0.005 Hz in (**a**) strain, ε, and in (**b**) stress, σ, against time, t, at potential range, E (dotted line, 1.0 to −0.55 V) of two subsequent cycles (3rd and 4th). The strain, ε, is shown in (**c**) and the stress differences, Δσ, in (**d**) against the charge density upon oxidation, Q_ox_. Negative strain as shown in (**c**) refers to the main expansion upon reduction and positive strain to expansion upon oxidation. The dashed lines in (**c**) represent the linear fit and are shown for orientation only.

To investigate the linear actuation response of PPy/DBS-PT films, square wave potential steps were performed at a frequency range of 0.0025 to 0.1 Hz. The strain and stress profiles at a frequency of 0.005 Hz are presented in Figure 5a,b, respectively. The current density time curves upon oxidation/reduction (shown for a frequency of 0.005 Hz in Figure S4a) over integration will yield the charge density. The strain and stress responses against charge density upon oxidation are presented in Figure 5c,d. The strain and stress responses against applied frequencies are shown in Figure S4b,c, respectively.

The strain and stress profiles (Figure 5a,b) have mixed-ion actuation of PPy/DBS-PT films, similar to those shown in Figure 4a,b. For PTA concentrations of PPy/DBS-PT films of 0.005 and 0.01 M, main expansion was found upon oxidation, while the PTA concentration of 0.05 M showed main expansion upon reduction. The stress profile in Figure 5b revealed that upon oxidation, stress increased and then decreased to the switching points of oxidation/reduction, with a strong increase and a further decrease (in case of 0.05 M PTA, the actuation direction was shown as the opposite). The main reason for such phenomena are mixed-ion movements [43], while upon oxidation, PF_6_^−^ anions moved in, simultaneously, TBA^+^ cations moved out (decrease of stress upon oxidation). If comparing the stress differences against frequencies (Figure S4c), there was a maximum of stress at 0.01 Hz for PPy/DBS-PT films (0.005 and 0.01 M), which refers to a decrease of cations’ influence upon reduction (with higher frequency, the stress shifts to mainly anion-dominated actuation). The main reason relies on the ion diffusion process having a shorter time at higher frequencies [45], where the anions incorporated upon oxidation cannot enter deeper cavities of PPy films compared to a lower frequencies. Consequently, less PF_6_^−^ are immobile in deeper pores, and upon reduction, the influence of cation incorporation (expansion at a reduction) decreases.

Comparing the strain against charge density (Figure 5c) revealed that with increasing charge density, the strain had nearly a linear response, referring to the faradaic process [46] wherein the charge density determines the linear actuation [47]. In the case of stress differences against charge density (Figure 5d), there was some odd behavior, with first and increase of stress and then a decrease of stress at a higher charge density. The stress maximum for PPy/DBS-PT (0.005 and 0.01 M PTA) was found at 0.01 Hz (Figure S4c). In the case of PPy/DBS-PT with 0.05 M PTA, the stress minimum was reflected at the same frequency. The explanation for such phenomena relies on mixed-ion movement during redox reaction. At low frequencies, dual-ion movements are prominent, while at higher frequencies, there is mainly one type of ion: either anions or cations are moving in or out, leading to an increase of stress. Table 2 compares the strain, stress, and charge density at the oxidation of PPy/DBS-PT films at frequencies of 0.0025 and 0.01 Hz.

**Figure 6:**
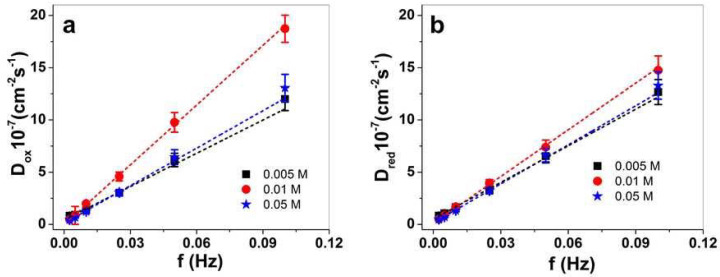
PPy/DBS-PT films with 0.005 M PTA (■), 0.01 M PPTA (●), and 0.05 M PTA (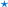), showing diffusion coefficients upon oxidation, D_ox_, in (**a**) and diffusion coefficients upon reduction, D_red_, in (**b**) against applied frequency at 0.0025–0.1 Hz. The dashed lines represent the linear fit and are shown for orientation only.

The charge density (Table 2) of PPy/DBS-PT films increased slightly with a higher concentration of PTA, as shown in Figure 5d. The best strain of 12.8% (0.0025 Hz frequency) in this study was found for PPy/DBS-PT (0.01 M), followed by PPy/DBS-PT (0.005 M) with 5.8% strain. The PPy/DBS-PT (0.05 M) film had negative strain at the same frequency, in the range of −4.8% (opposite actuation direction). The 0.01 M PTA had the best stress for PPy/DBS-PT films, followed by 0.005 M PTA, with the lowest stress found for 0.05 M PTA. The diffusion coefficients regarding Equations (1) and (2) were determined, with results presented in Figure 6a,b.

Figure 6a,b show a general tendency of the diffusion coefficients increasing with increasing frequency. This relies on different processes taking place either upon oxidation or reduction of the PPy/DBS-PT films regarding the electrochemically stimulated conformational relaxation (ESCR) model [23,48]. At low frequency, the longer time leads to the diffusion of ions inside or outside the films to relaxation/swelling or compaction/shrinking, so-called structural and conformational changes [49]. Only swelling or shrinking took place at shorter times (higher frequency). The PPy/DBS-PT (0.01 M) stood out (also found in former work with aqueous electrolytes [14]), especially upon oxidation (Figure 6a), showing much higher diffusion coefficients (~1.5 times). At a frequency of 0.1 Hz, the PPy/DBS-PT film with 0.01 M PTA had diffusion coefficients upon oxidation of 18.7 ± 1.3 10^−7^ cm^−2^s^−1^, in comparison to 0.005 and 0.05 M PTA which were in the range of 12–13 ± 1.1 10^−7^ cm^−2^s^−1^. The differences in diffusion coefficients upon reduction (Figure 6b) were not so pronounced, with nearly 1.1 times higher values (0.1 Hz) of the PPy/DBS-PT (0.01 M) film. At a frequency of 0.1 Hz, the PPy/DBS-PT films with 0.005 and 0.01 M PTA concentrations only had expansion upon oxidation, and neglected expansion upon reduction. Therefore, only PF_6_^−^ anions moved in upon oxidation and moved out upon reduction. In the case of 0.05 M PTA, the expansion upon reduction of PPy/DBS-PT films referred to TBA^+^ cations’ ingress to balance the negative charges of PF_6_^−^ anions that stayed immobile in the film; therefore, upon oxidation, the TBA^+^ cations left the film.

In summary, the PPy/DBS-PT film with 0.01 M PTA stood out because it had the best strain and stress, the highest diffusion coefficients, the best electronic conductivity after actuation, and the highest decrease in elastic modulus after actuation (Table 1), making this PTA concentration ideal for linear actuators, with possible applications either in soft robotics or smart textiles.

### 3.3. Energy Storage

Flexible materials that can provide energy storage have been the focus of research in recent decades, with envisaged multifunctional purposes [50,51]. In general, carbon-related materials are applied due to their high capacitance and mesoporous characteristics, while conducting polymers are pseudo-capacitors mostly applied in combination with carbon materials [52] or with inorganic salts such as POM [10]. POM is known to have a fast faradaic process, supporting either non-faradaic materials such as CNT, with specific capacitance found in the range of 40 F g^−1^ (in H_2_SO_4_) [53], or in hydrogels (PMo_12_) applied in the aqueous electrolyte, reaching up to 300 F g^−1^ [54]. Other reports using conducting polymers such as Cobalt(II)phthalocyanine complexes [55] showed specific capacitance in the aqueous electrolyte in the range of 11 F g^−1^. A combination of PPy/DBS with PTA reported from former work [3] had optimal specific capacitance for PPy/DBS-PT (0.01 M) in the aqueous electrolyte in the range of 223 F g^−1^ (0.09 A g^−1^) [14].

**Figure 7:**
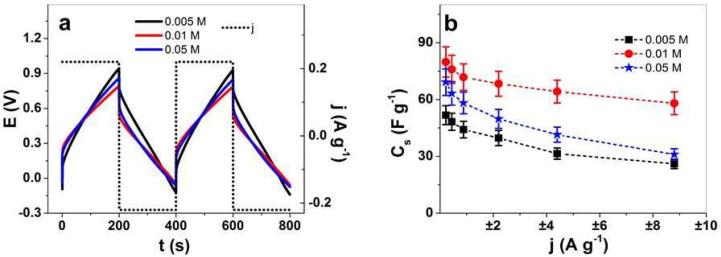
Chronopotentiometric measurements in the TBAPF_6_-PC electrolyte of PPy/DBS-PT films with PTA concentrations of 0.005 M (black line, ■), 0.01 M (red line, ●), and 0.05 M (blue line, 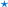). The potential time curves at applied current density of ±0.22 A g^−1^ (0.0025 Hz) of PPy/DBS-PT films (two subsequent cycles 3rd and 4th) are shown in (**a**). The specific capacitance, C_s_, against applied current densities, j (±0.22 A g^−1^ to ±8.8 A g^−1^, 0.0025–0.1 Hz, having the same charge density of ±44 C g^−1^), is presented in (**b**).

Here, we investigate the specific capacitance of PPy/DBS-PT films to find out which are the best for flexible energy storage devices in an organic electrolyte. Chronopotentiometric measurements of PPy/DBS-PT films showed the potential time curves at ±0.22 A g^−1^ (0.0025 Hz), as shown in Figure 7a, and those from PPy/DBS are shown in Figure S5a at a current density of ±0.21 A g^−1^. From Equation (3), the specific capacitance of PPy/DBS-PT films was calculated, and the results are presented in Figure 7b, and those from pristine PPy/DBS films are shown in Figure S5b.

The potential time curves in Figure 7a revealed minor differences in the chronopotentiogram, with slightly higher voltage evolution at charging (0.95 V) for PPy/DBS-PT at 0.005 M PTA, followed by 0.05 M (0.85 V), and the lowest was found for 0.01 M PTA (0.78 V). The potential time curves (Figure 7a) at each PTA concentration confirmed that the charging/discharging of PPy/DBS-PT films were in balance [38]. The specific capacitance obtained from Equation (3) at each applied current density for PPy/DBS-PT films (Figure 7b) revealed that the best specific capacitance at ±0.22 A g^−1^ was found for PPy/DBS-PT with 0.01 M PTA at 80 ± 7.5 F g^−1^, followed by 0.05 M PTA at 69.2 ± 6.4 F g^−1^. The lowest specific capacitance in the range of 51.8 ± 4.9 F g^−1^ was found for PPy/DBS-PT 0.005 M PTA films, with a similar size as pristine PPy/DBS, 51.2 ± 3.2 F g^−1^ (Figure S5b). The specific capacitance of PPy in an organic (acetonitrile) electrolyte [56] was reported to be in the range of 14 F g^−1^, and recently, PPy combined with polymerized ionic liquids [57] showed a specific capacitance at 75 F g^−1^. The application of PPy/DBS-PT films in organic electrolytes has, in general, a lower capacitance than those in aqueous solution [6]. PPy combined with POM (Lindqvist-type anions, V_n_M_6-n_O_19_^(2+n)−^) showed specific capacitance in an organic electrolyte (acetonitrile) in the range of 25–36 F g^−1^ [7], and research on PPy/DBS-PT (0.01 M) in a NaClO_4_-PC electrolyte had specific capacitance in the range of 34 F g^−1^ (0.05 A g^−1^) [20]. In summary, the inclusion of PTA in PPy/DBS films led to enhanced specific capacitance, with an optimum found for 0.01 M PTA, which enables the use of these composites in flexible fabrics where energy storage is required.

## 4. Conclusions

PPy/DBS-PT films with different PTA concentrations in electropolymerization were studied in this work, revealing that with increasing concentrations of PTA (from 0.005 to 0.05 M), the films became dense and compact. Linear actuation of these composites was studied in the TBAPF_6_-PC electrolyte, showing that a higher PTA concentration has an effect of enlarging the expansion upon reduction of the observed mixed-ion actuations. For PPy/DBS-PT (0.05 M PTA), main expansion was found upon reduction, with a strain of 4.8% and stress of 0.1 MPa, while the other PPy/DBS-PT films showed main expansion upon oxidation. The best linear actuation, accompanied by 1.5 times higher diffusion coefficients in strain of 12.8% and stress in the range of 0.65 MPa, was found for PPy/DBS-PT (0.01 M) films. The incorporation of redox-active PTA molecules also influenced the specific capacitance, which was found optimal for PPy/DBS-PT (0.01 M) films in the range of 80 F g^−1^ (±0.22 A g^−1^), revealing that this PTA concentration was most advantageous, with possible applications of these composites in dual functionality of actuators and energy storage.

## Figures and Tables

**Table 1:** Electronic conductivity and elastic modulus of PPy/DBS-PT films before and after actuation.

PPy/DBS-PT PTA Concentration	Conductivity (S cm^−1^)		Elastic Modulus (MPa)	
	BA	AA	BA	AA
0.005 M	7.4 ± 0.7	9.5 ± 0.8	2.45 ± 0.12	1.61 ± 0.12
0.01 M	9.7 ± 0.8	14.4 ± 1.3	1.54 ± 0.18	0.39 ± 0.03
0.05 M	11.3 ± 0.9	10.2 ± 0.9	0.82 ± 0.07	0.67 ± 0.05

**Table 2:** PPy/DBS-PT films (different PTA concentrations), showing strain, ε, stress differences, Δσ, and charge density upon oxidation, Q_ox_, at frequencies of 0.0025 and 0.01 Hz.

PPy/DBS-PT PTA (M)	Q_ox_ (C cm^−3^)		ε (%)		Δσ (MPa)	
	0.0025 Hz	0.01 Hz	0.0025 Hz	0.01 Hz	0.0025 Hz	0.01 Hz
0.005	94.3 ± 8.1	32.5 ± 2.7	5.8 ± 0.5	2.5 ± 0.23	0.21 ± 0.02	0.58 ± 0.05
0.01	100.1 ± 9.2	34.4 ± 3.1	12.8 ± 1.1	5.0 ± 0.44	0.36 ± 0.03	0.65 ± 0.06
0.05	107.0 ± 9.8	35.7 ± 3.3	−4.8 ± 0.4	−1.8 ± 0.13	0.15 ± 0.01	0.1 ± 0.01

## Data Availability

The data presented in this study are available on request from the corresponding author.

## Supplementary Materials

The following supporting information can be downloaded at: https://www.mdpi.com/article/10.3390/ma15103619/s1, Figure S1. SEM surface and cross section image (scale bar 10 μm) of pristine PPy/DBS. Figure S2. EDX spectroscopy of PPy/DBS-PT films in cross-section image after polymerization and washing steps in oxidized state (1.0 V) in different PTA concentrations of 0.005 M (black line), 0.01 M (red line) and 0.05 M (blue line). Figure S3. Cyclic voltammetry (scan rate 5 mV s^−1^, 3rd cycle) of pristine PPy/DBS films (black line) in TBAPF_6_-PC electrolyte are showing in a: strain, in b: stress, in c: current density and in d: charge density curves against potential E (1.0 V to −0.55 V). The arrows symbolize the start and ending point of the cycle. Figure S4. Square potential steps of PPyPOM films in TBAPF_6_-PC electrolyte at different POM concentration 0.005 M (black line, ■), 0.01 M (red line, ●) and 0.05 M (blue line, ) presents current density time curves at 0.0025 Hz at two subsequence cycles (3rd–4th) at potential range E (dotted line, 1.0 V to −0.55 V) in (a). The strain ε is presented in (b) and the stress differences Δσ against applied frequencies f (0.0025–0.1 Hz) are shown in (c). Figure S5. Pristine PPy/DBS films (black line, ■) in chronopotentiometric measurements in TBAPF_6_-PC electrolyte showing in a: the potential time curve of two subsequent cycles (3rd–4th) at applied current density ± 0.212 A g^−1^. The specific capacitance C_s_ against the current densities (± 0.212 A g^−1^, ± 0.424 A g^-1^, ± 0.848 A g^−1^, ± 2.12 A g^−1^, ± 4.24 A g^−1^ and ± 8.48 A g^−1^, having same charge densities of ± 42.4 C g^−1^) are presented in (b).
