# Supplementary material for: Role of Polyoxometalate Contents in Polypyrrole: Linear Actuation and Energy Storage
Source: Materials (Basel). 2022 May 18;15(10):3619. doi: 10.3390/ma15103619 (PMC9145510; doi:10.3390/ma15103619)
Supplement: Supplementary file 1 [file materials-15-03619-s001.zip › materials-1672603-supplementary.pdf]

Supplementary

Role of Polyoxometalate Contents in Polypyrrole: Linear Actuation and Energy Storage

Quoc Bao Le [1], Zane Zondaka [2], Madis Harjo [2], Ngoc Tuan Nguyen [3] and Rudolf Kiefer [1,*]

[1] Conducting Polymers in Composites and Applications Research Group, Faculty of Applied Sciences, Ton Duc Thang University, Ho Chi Minh City 700000, Vietnam; lequocbao@tdtu.edu.vn
[2] Intelligent Materials and Systems Lab, Faculty of Science and Technology, University of Tartu, Nooruse 1, 50411 Tartu, Estonia; zane.zondaka@ut.ee (Z.Z.); madis.harjo@gmail.com (M.H.)
[3] Faculty of Applied Sciences, Ton Duc Thang University, Ho Chi Minh City 700000, Vietnam; nguyenngoctuan@tdtu.edu.vn
* Correspondence: rudolf.kiefer@tdtu.edu.vn; Tel.: +886-905-605-515

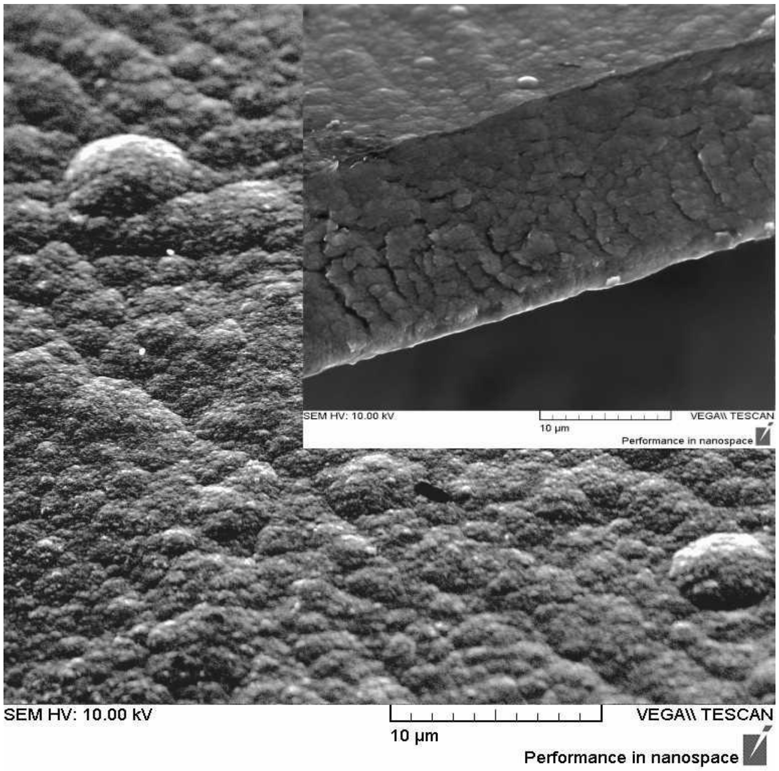

Figure S1. SEM surface and cross section image (scale bar 10 μm) of pristine PPy/DBS.

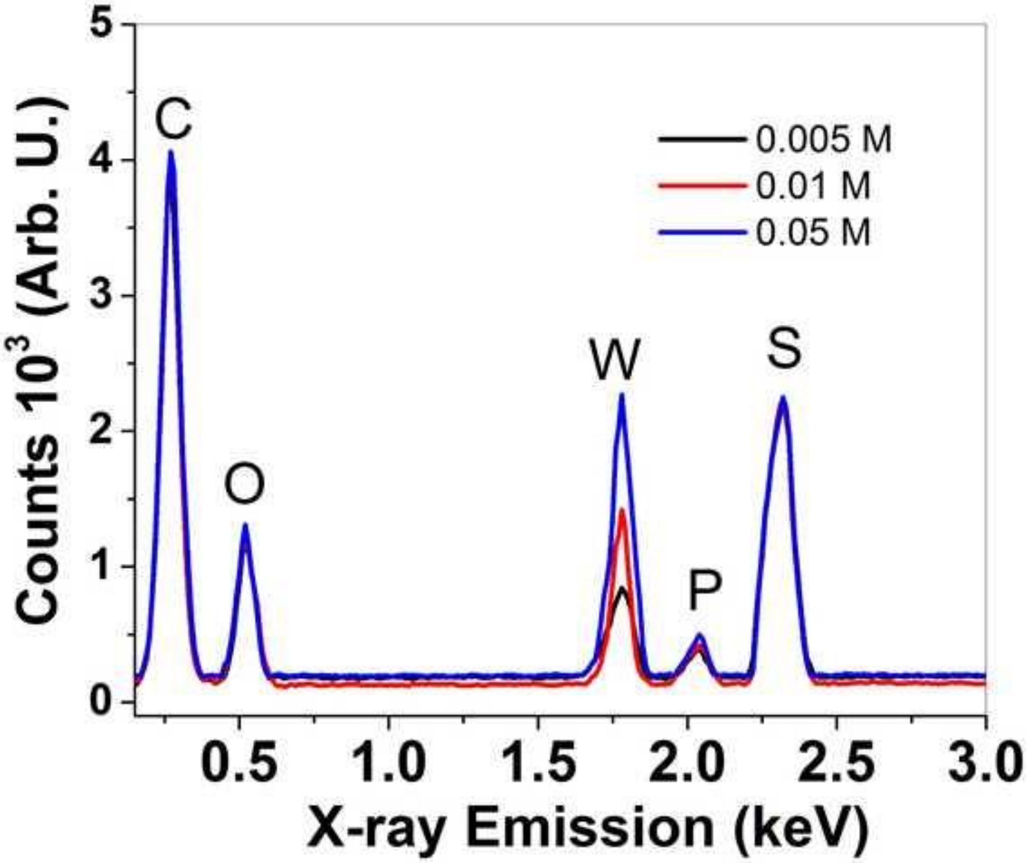

Figure S2. EDX spectroscopy of PPy/DBS-PT films in cross-section image after polymerization and washing steps in oxidized state (1.0 V) in different PTA concentrations of 0.005 M (black line), 0.01 M (red line) and 0.05 M (blue line).

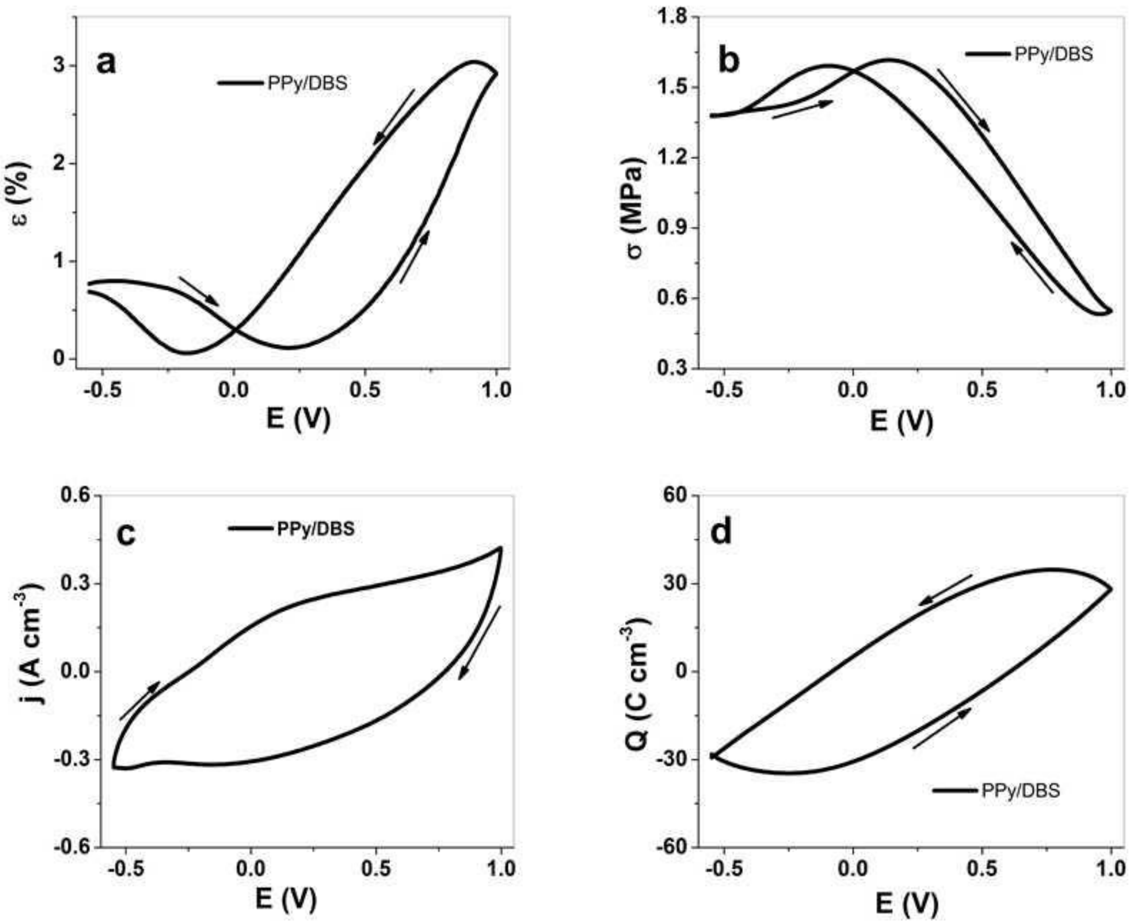

Figure S3. Cyclic voltammetry (scan rate 5 mV s^{-1}, 3rd cycle) of pristine PPy/DBS films (black line) in $TBAPF_6$-PC electrolyte are showing in a: strain, in b: stress, in c: current density and in d: charge density curves against potential E (1.0V to -0.55V). The arrows symbolize the start and ending point of the cycle.

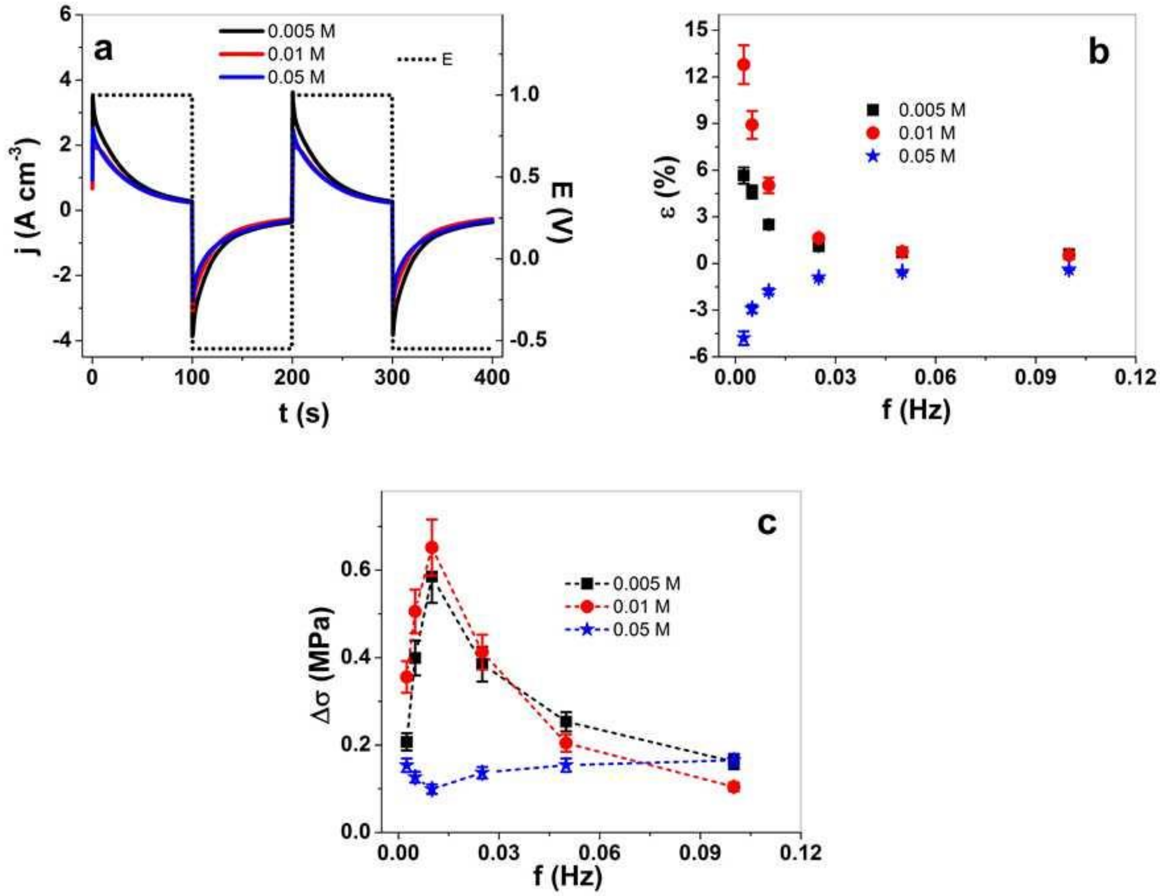

Figure S4. Square potential steps of PPyPOM films in $TBAPF_6$-PC electrolyte at different POM concentration 0.005 M (black line, ■), 0.01 M (red line, ●) and 0.05 M (blue line, ★) presents current density time curves at 0.0025 Hz at two subsequence cycles (3rd -4th) at potential range E (dotted line, 1.0V to -0.55V) in a). The strain ε is presented in b) and the stress differences Δσ against applied frequencies f (0.0025 Hz – 0.1 Hz) are shown in c).

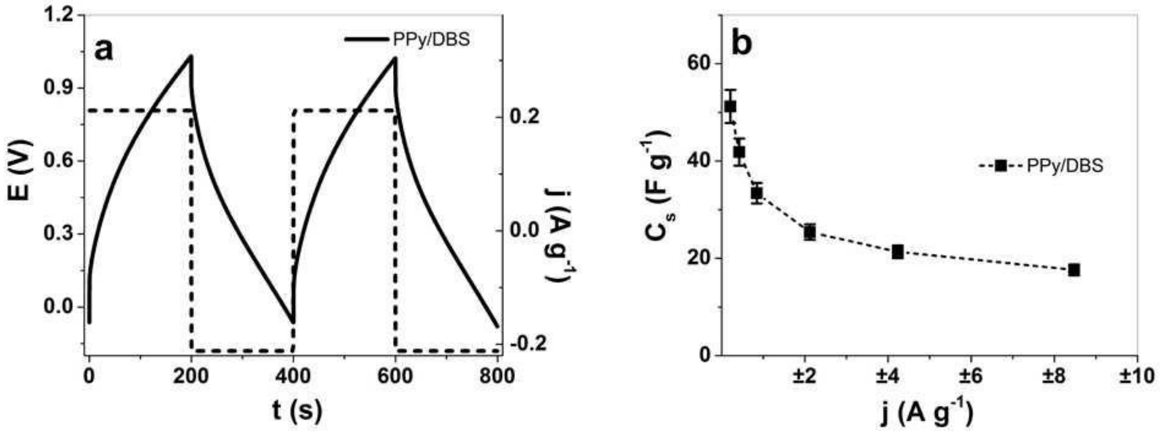

Figure S5. Pristine PPy/DBS films (black line, ■) in chronopotentiometric measurements in $TBAPF_6$-PC electrolyte showing in a: the potential time curve of two subsequent cycles (3rd – 4th) at applied current density ± 0.212 A g^{-1}. The specific capacitance C_s against the current densities (± 0.212 A g^{-1}, ± 0.424 A g^{-1}, ± 0.848 A g^{-1}, ± 2.12 A g^{-1}, ± 4.24 A g^{-1} and ± 8.48 A g^{-1}, having same charge densities of ± 42.4 C g^{-1}) are presented in b).
